# Optimal Conservation of Migratory Species

**DOI:** 10.1371/journal.pone.0000751

**Published:** 2007-08-15

**Authors:** Tara G. Martin, Iadine Chadès, Peter Arcese, Peter P. Marra, Hugh P. Possingham, D. Ryan Norris

**Affiliations:** 1 Centre for Applied Conservation Research, Forest Sciences, University of British Columbia, Vancouver, British Columbia, Canada; 2 INRA, UR875, Unité de Biométrie et Intelligence Artificielle, Toulouse, France; 3 Smithsonian Migratory Bird Center, National Zoological Park, Washington, D. C., United States of America; 4 The Ecology Centre and Department of Mathematics, University of Queensland, Queensland, Australia; 5 Department of Integrative Biology, University of Guelph, Guelph, Ontario, Canada; University of Edinburgh, United Kingdom

## Abstract

**Background:**

Migratory animals comprise a significant portion of biodiversity worldwide with annual investment for their conservation exceeding several billion dollars. Designing effective conservation plans presents enormous challenges. Migratory species are influenced by multiple events across land and sea–regions that are often separated by thousands of kilometres and span international borders. To date, conservation strategies for migratory species fail to take into account how migratory animals are spatially connected between different periods of the annual cycle (i.e. migratory connectivity) bringing into question the utility and efficiency of current conservation efforts.

**Methodology/Principal Findings:**

Here, we report the first framework for determining an optimal conservation strategy for a migratory species. Employing a decision theoretic approach using dynamic optimization, we address the problem of how to allocate resources for habitat conservation for a Neotropical-Nearctic migratory bird, the American redstart *Setophaga ruticilla*, whose winter habitat is under threat. Our first conservation strategy used the acquisition of winter habitat based on land cost, relative bird density, and the rate of habitat loss to maximize the abundance of birds on the wintering grounds. Our second strategy maximized bird abundance across the entire range of the species by adding the constraint of maintaining a minimum percentage of birds within each breeding region in North America using information on migratory connectivity as estimated from stable-hydrogen isotopes in feathers. We show that failure to take into account migratory connectivity may doom some regional populations to extinction, whereas including information on migratory connectivity results in the protection of the species across its entire range.

**Conclusions/Significance:**

We demonstrate that conservation strategies for migratory animals depend critically upon two factors: knowledge of migratory connectivity and the correct statement of the conservation problem. Our framework can be used to identify efficient conservation strategies for migratory taxa worldwide, including insects, birds, mammals, and marine organisms.

## Introduction

Migratory birds comprise more than 80% of avian diversity in temperate regions of the world [1]. Protecting these species presents a unique conservation challenge because population abundance is influenced by geographically separated events that occur during different periods of the year [2]. If maximizing population persistence is a central goal of species conservation [3], then the optimal allocation of resources for conserving migratory species should consider population dynamics throughout the annual cycle, not just during a single period of the year. However, the lack of information on how populations are geographically linked between periods of the annual cycle (i.e. migratory connectivity) has made it virtually impossible to develop conservation strategies that incorporate year-round dynamics [4].

Investment in migratory bird conservation is substantial with over US$650 million allocated to wetland lease and acquisition in North America for migratory birds in 2005 alone [5]. Global estimates for this same fiscal year are likely to be several billion if all migratory species are considered [6]. Current strategies used to allocate funds are ad-hoc or based on ranking methods [7] and fail to incorporate both migratory connectivity and the cost of implementing the conservation action.

Several recent studies addressing cost-effectiveness in conservation planning provide guidance on how to estimate where, when and how much to invest for the conservation of non-migratory species [7]–[10]. We build upon these approaches using a unique dataset from a long-distance migratory songbird, the American redstart (*Setophaga ruticilla)*, to examine whether incorporating migratory connectivity into habitat protection schemes influences decisions designed to maximize the persistence of populations. We demonstrate for migratory species how failure to include estimates of migratory connectivity leads to the improper formulation of the problem and could doom regional populations to near extinction.

## Results

Following a decision theoretic framework [7], [11], [12], we employed an optimal search algorithm Dijkstra [13] to find optimal resource allocation strategies for two problems; first, to maximize the number of birds protected across the wintering range, and second, to maximize the number of birds protected across the entire range of the species by adding the constraint of maintaining a minimum population size within each of five temperate breeding regions (see Materials and Methods).

**Figure 1:**
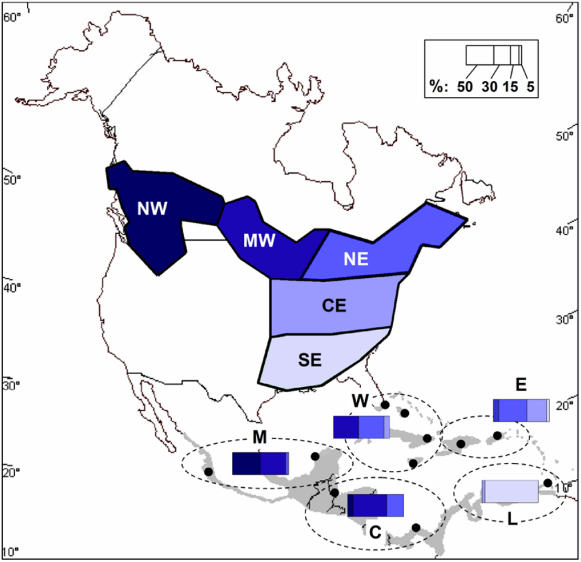
Patterns of connectivity in American redstarts. The distribution of the most likely breeding region (NW, Northwest; MW, Midwest; NE, Northeast; CE, Central-east; SE, Southeast) for individuals at each wintering region (M, Mexico; C, Central America; W, Western Greater Antilles; E, Eastern Greater Antilles; L, Lesser Antilles/South America). Black dots indicate sampling locations and bars indicate the proportion of individuals assigned to each breeding region (rounded to the nearest 5%) [adapted from ref 14].

To solve the first problem, we developed an optimization model where sequential decisions to acquire parcels of wintering habitat, within a fixed budget and time period, were based on rates of winter habitat loss, land value, and the relative density of birds in each wintering region (Table 1; see Supporting Information, Text S1 i, ii). To solve the second problem, we developed an optimization model that incorporated information on migratory connectivity, using stable-hydrogen isotopes (δD) to estimate the breeding origin of individuals sampled on the tropical wintering grounds [14] (Figure 1; Supporting Information, Table S1). This information allowed us to estimate the relative change in abundance of breeding populations as a result of habitat conservation on the wintering grounds. Although we used land acquisition as our mechanism to preserve populations, our models can readily incorporate ongoing management costs [15] and other conservation strategies such as easements or active management [10].

**Table 1:** Estimates used to parameterize optimization problem.

Region	Bird density (km^−2^)	Habitat Cost ($US millions/km^2^)	Cost per bird ($US)	Annual Rate of habitat loss	Available habitat (km^2^)	Parcel size (km^2^)	Parcels per region
Western Greater Antilles	360 (2)	2.88 (4)	8,012	2.5% (1)	351	43	8
Eastern Greater Antilles	537 (1)	3.88 (5)	7,238	1.4% (3)	3523	252	13
Mexico	215 (4)	2.29 (3)	10,645	2.1% (2)	4400	462	9
Lesser Antilles/South America	320 (3)	1.85 (2)	5,768	0.7% (4)	2366	76	30
Central America	90 (5)	0.63 (1)	7,055	0.7% (4)	2207	77	28

Bird density, cost, threat, available habitat, parcel size and number of parcels available for acquisition for 5 regions in the wintering range of the American redstart. Numbers in brackets represent a rank of parameters based on their relative priority from 1 (high priority) to 5 (low priority). Based on these ranks it is impossible to design an optimal investment strategy.

**Figure 2:**
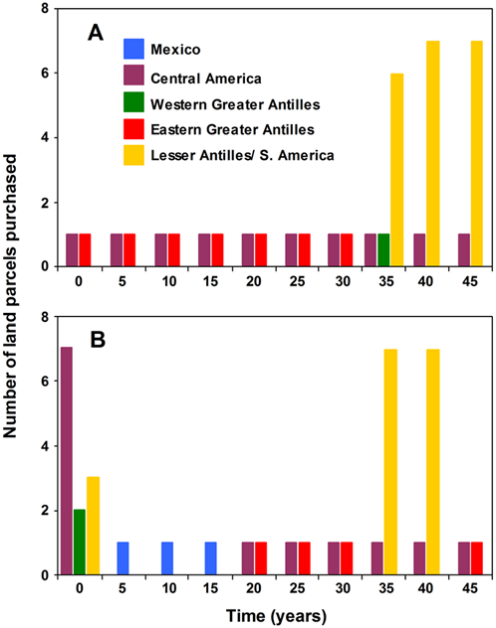
The total number of parcels purchased in each region over a 45-year time-horizon. When the objective is to (a) maximize the number of birds on the wintering grounds and (b) maximize the number of birds on the wintering grounds and protect a minimum of 30% of birds in each breeding region by taking migratory connectivity into account.

Our first objective was to maximize the number of birds throughout the wintering range. The optimal resource allocation strategy for this objective was to invest solely in Central America and the Eastern Greater Antilles for the first 30 years (Figure 2a), then include a single investment in the Western Greater Antilles at year 35, and a heavy investment in the Lesser Antilles/South America through to year 45. This optimal strategy did not recommend an investment in Mexico.

In contrast, by adopting an objective that included the protection of at least 30% of the redstarts in each of five breeding regions through incorporating information on migratory connectivity, we obtained an entirely different conservation strategy (Figure 2b). In the first five years, investments were targeted in Western Greater Antilles, the Lesser Antilles/South America, and heavily in Central America. In years 5–20, investments were made solely in Mexico with one land parcel purchased every 5 years. In years 20–45, investments were made in Central America and the Eastern Greater Antilles, and heavily in the Lesser Antilles/South America after year 35.

**Figure 3:**
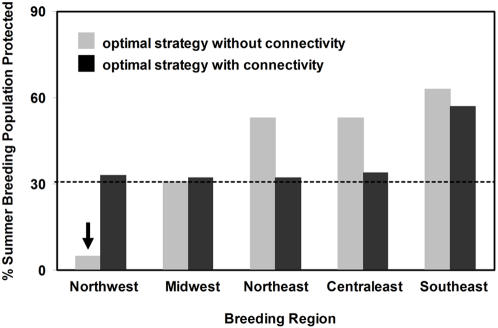
The proportion of the summer breeding population protected, through the conservation of habitat parcels in the wintering regions with an optimal strategy that ignores migratory connectivity and one that incorporates migratory connectivity. The Northwest population is reduced to less than 2%, well below the 30% threshold, when connectivity data are ignored as denoted by the arrow.

The conservation strategies to achieve each objective resulted in large differences in the proportion of birds protected on the breeding grounds (Figure 3). When migratory connectivity was ignored, the Northwest region shrank to less than 2% of its original population size because 82% of birds breeding in this region wintered in Mexico [14], which did not receive any protection under objective 1. In contrast, incorporating the conservation of regional breeding populations into the decision analysis (objective 2) resulted in the purchase of habitat in Mexico despite its cost.

## Discussion

We have discovered that key decisions aimed at conserving migratory populations may depend critically on considering the link between different periods of the annual cycle. Focusing solely on maximizing winter population size without considering their connection to breeding areas has the potential to reduce dramatically the regional distribution of redstart populations in North America. In contrast, using information on migratory connectivity allowed us to set targets for maintaining minimum population sizes within each breeding region, thereby dramatically changing the decision pathways and improving the regional stability thousands of kilometers away on breeding areas.

Our results have immediate implications for the allocation of funds aimed at conserving migratory species. We demonstrate that using a decision theoretic approach integrated with information on migratory connectivity will improve the efficiency of resource allocation. This approach is applicable to migratory species around the globe and offers an effective means of identifying and prioritizing conservation investment strategies.

Due to the number and complexity of parameters involved, it is unlikely that the strategies we found to be optimal would arise from a ranking-based method (see Table 1) or expert opinion. We also suggest that the cost of protecting the wrong or insufficient winter habitat, which may result in the loss of regional breeding populations, is very likely to exceed the cost of collecting information on migratory connectivity and running analyses like those described here. A decision theoretic approach incorporating species density, level of threat and cost of collecting migratory connectivity per species, could be used to develop a species prioritization list for the collection of data on migratory connectivity. The range of many migratory species also spans multiple countries or even continents. The probability of compliance with conservation strategies will likely vary by country or region and should be considered as another potential factor in the decision analysis. For migratory birds in particular, the inclusion of key information about stopover regions including level of threat, the number of individuals that use each area, their origin and destination will also be extremely valuable input for this type of decision analysis.

Because our aim was to find an optimal solution, the sheer size of the solution search space (see Supporting Information, Text S1 iii) forced us to assume that the system dynamics were deterministic. In doing so, we made several simplifying assumptions. In the absence of quantitative data, we assumed that American redstart density was directly proportional to habitat loss, whereby a loss in a habitat parcel resulted in the loss of birds occupying that parcel. The budget per time-step was based on the cost of a parcel in the most expensive region. The number of parcels that could be purchased for the equivalent cost in each other region determined the number of parcels available for purchase. This alleviated the need for a stochastic process to determine how to allocate leftover funds at each time-step (See Supporting Information, Text S1). We acknowledge that species density, rate of habitat loss, and cost are subject to unknown stochastic processes. However, it is not clear whether a suboptimal stochastic solution [7] would be more effective than our optimal deterministic solution. We also note that the optimal strategy presented here is only for one species, whereas managers must often make decisions that are designed to benefit multiple species [16]. Our study introduces a framework for achieving this goal while emphasizing the importance of considering multiple periods of the annual cycle. We also show that the optimal decision schedule depends on the time-horizon for planning such strategies (Supporting Information: Text S1 iv, Figure S1). Recognizing this dependency will be critical when making decisions with fixed, short-term budgets subject to long-term uncertainty.

Despite these caveats, we demonstrate the over-riding importance of considering year-round dynamics for allocating conservation funds to habitat acquisition for migratory species. Understanding how different periods of the year are influenced by conservation strategies can only be achieved through information on migratory connectivity. Our modeling framework incorporates this factor and can be applied to all migratory taxa. With this knowledge, adopting a decision theoretic approach to the optimal allocation of conservation resources should provide an effective and timely method for securing the persistence and future of migratory species around the world.

## Materials and Methods

### Study Species

American redstarts are small (8 g) migratory songbirds that breed in deciduous and mixed deciduous-coniferous forest across North America and winter in Central America, the Caribbean, and the north coast of South America [17]. Evidence suggests that the quality of habitat used by redstarts on the tropical wintering grounds influences individual success. Redstarts occupying high-quality mesic habitats, such as coastal mangroves and lowland forest, are in better condition over the winter, have higher annual survival rates, depart earlier for spring migration, and have higher reproductive success the following season on the temperate breeding grounds compared to individuals occupying lower quality habitats, such as tropical dry forests [18]–[21]. These findings suggest that the availability of high quality winter habitat is a key limiting factor to the abundance of redstarts. In this study, we consider coastal mangroves only as they are considered to be most limiting non-breeding habitat for this species [18]–[21].

### Problem definition and approach

In the first problem our goal was to maximize the number of birds protected across the winter range. In doing so, we took into account the amount of winter habitat available for conservation, its rate of loss, the cost of land acquisition, and estimates of redstart density across the winter range (Table 1). In the second problem our goal was to maximize the number of birds protected across its entire range by adding the constraint of maintaining a minimum proportion (≥30%) of each regional breeding population (Supporting Information, Text S1 i). To do this, we incorporated information on migratory connectivity to estimate the proportion of birds at a given wintering site that bred in one of five breeding regions in North America [14] (Figure 1; Supporting Information, Table S1). The winter regions are defined as the Western Greater Antilles (Dominican Republic, Haiti, Puerto Rico), Eastern Greater Antilles (Florida, Bahamas, Jamaica), Lesser Antilles/South America (Trinidad, Tobago, Venezuela), Mexico, and Central America (Belize, Panama) (Figure 1).

### Algorithm

We implemented an optimal search algorithm, ‘Dijkstra’ [22], to find the shortest path from our starting state (the current situation) to our goal state. Dijkstra's algorithm does not traverse the entire solution space but rather only pursues paths that are likely to lead to the optimal path and is therefore an efficient and computationally fast method of finding an optimal solution for problems of this size (Supporting Information, Text S1 iii).

The efficacy of our optimal search algorithm was evaluated through a comparison of results with a second algorithm ‘myopic’ [7] (see Supporting Information, Text S1 iv). This heuristic algorithm makes the optimal choice one step at a time with the goal of finding a global optimum. In other words, at each time step, the site with the most birds per unit cost is chosen. However, because this algorithm moves in a single forward direction, it is ‘short-sighted’ leading to solutions that are likely to be suboptimal (cf. the maximize ‘short-term gain’ and ‘minimize short-term loss’ heuristics [7]). We found that ‘Dijkstra’ outperformed the ‘myopic’ algorithm returning between 4 to 28 percent more birds protected per dollar spent depending on the time-horizon. Our findings are presented for Dijkstra's algorithm using a 45-year time-horizon.

### Simulations and Sensitivity

We ran scenarios with each algorithm for each objective function, with various finite time-horizons (5 to 60 years). We determined the sensitivity of changes in cost and migratory connectivity by comparing our results to simulations run with an equal cost function for each region. This comparison revealed that including information on migratory connectivity resulted in greater differences in the prioritization of winter parcels than regional variation in cost of winter habitat. All models and simulations were run using MATLAB version 7.0 [23].

## Supporting Information

Text S1 (0.07 MB DOC)

Table S1 American redstart migratory connectivity. The proportion of birds that migrate from a winter region to each breeding region based on stable-hydrogen isotopes in feathers (Figure 1, see ref [1]). NW  =  Northwest; MW  =  Midwest; NE  =  Northeast; CE  =  Central-east; SE  =  Southeast.
(0.04 MB DOC)

Figure S1 Contrasting the performance of two algorithms ‘myopic’ and Dijkstra over different time-horizons (5 to 60 years) showing the total number of birds saved when the objective function is to maximize the number of birds in the winter population.
(0.08 MB TIF)

## References

[1] Rappole JH (1995). The Ecology of Migrant Birds: A Neotropical Perspective..

[2] Webster MS, Marra PP, Haig SM, Bensch S, Holmes RT (2002). Links between worlds: unraveling migratory connectivity.. TREE.

[3] Williams PH, Araujo MB (2000). Using probability of persistence to identify important areas for biodiversity conservation.. Proceedings of the Royal Society London Series B.

[4] Marra PP, Norris DR, Haig SM, Webster M, Royle JA, Crooks K, Muttulingam S (in press). Migratory connectivity.. Maintaining Connections for Nature: Cambridge University Press, New York.

[5] Anon (2006). 2005 Annual Report-Migratory Bird Conservation Commission.. http://www.fws.gov/realty/pdf_files/2005%20MBCC%20Report.pdf.

[6] Anon (2003). Convention on the conservation of migratory species of wild animals (CMS).. http://www.cms.int/pdf/convtxt/cms_convtxt_english.pdf.

[7] Wilson KA, McBride MF, Bode M, Possingham HP (2006). Prioritizing global conservation efforts.. Nature.

[8] Costello C, Polasky S (2004). Dynamic reserve site selection.. Resource and Energy Economics.

[9] Davis FW, Costello C, Stoms D (2006). Efficient conservation in a utility-maximization framework.. http://www.ecologyandsociety.org/vol11/iss31/art33/.

[10] Wilson KA, Underwood EC, Klausmeier KR, Murdoch B, Reyers B (in press). Saving the world's Mediterranean ecoregions: what to do and where?. PLoS Biology.

[11] Possingham HP, Andelman SJ, Noon BR, Trombulak S, Pulliam HR, Soule ME, Orians GH (2001). Making smart conservation decisions.. Research priorities for nature conservation.

[12] Haight RG, Cypher B, Kelly PA, Phillips S, Ralls K (2004). Optimizing reserve expansion for disjunct populations of San Joaquin kit fox.. Biological Conservation.

[13] Cormen TH, Leiserson CE, Rivest RL, Stein C (2001). Introduction to Algorithms, Second Edition: MIT Press and McGraw-Hill..

[14] Norris DR, Marra PP, Kyser TK, Royle JA, Bowen GJ (2006). Migratory connectivity of a widely distributed Neotropical-Neartic migratory songbird.. Ornithological Monographs.

[15] Moore J, Balmford A, Allnutt T, Burgess N (2004). Integrating costs into conservation planning across Africa.. Biological Conservation.

[16] Nicholson E, Possingham HP (2006). Objectives for Multiple-Species Conservation Planning.. Conservation Biology.

[17] Sherry TW, Holmes RT, Poole A, Gill F (1997). American Redstart (*Setophaga ruticilla*), No. 277.. Birds of North America: The academy of Natural Sciences of Philadelphia, Philadelphia, Pennsylvania; the American Ornithologists' Union.

[18] Marra PP, Hobson KA, Holmes RT (1998). Linking winter and summer events in a migratory bird by using stable-carbon isotopes.. Science.

[19] Marra PP, Holmes RT (2001). Consequences of dominance-mediated habitat segregation in a migrant passerine bird during the non-breeding season.. Auk.

[20] Norris DR, Marra PP, Kyser TK, Sherry TW, Ratcliffe LM (2004). Tropical winter habitat limits reproductive success on the temperate breeding grounds in a migratory bird.. Proceedings of the Royal Society Series B.

[21] Studds CE, Marra PP (2005). Nonbreeding habitat occupancy and population processes: an upgrade experiment with a migratory bird.. Ecology.

[22] Cormen TH, Leiserson CE, Rivest RL, Stein C (2001). Dijkstra's algorithm. Introduction to Algorithms, Second Edition: MIT Press and McGraw-Hill..

[23] Mathworks (2002). MATLAB 7.0..

