# Supplementary material for: Optimal Conservation of Migratory Species
Source: PLoS One. 2007 Aug 15;2(8):e751. doi: 10.1371/journal.pone.0000751 (PMC1937026; doi:10.1371/journal.pone.0000751)
Supplement: Text S1 — (0.07 MB DOC) [file pone.0000751.s001.doc]

**Supporting Information: Text S1**

**Optimal Conservation of Migratory Species**

Tara G. Martin, Iadine Chadès, Peter Arcese, Peter P. Marra, Hugh P. Possingham, D. Ryan Norris

**i. Problem formulation**

We define the winter habitat of our migrant species within a finite set of winter regions *W={w_1_, …,w_i_, …,w_n_}*. Each winter region *w_i_* is characterized by a specific rate of habitat loss per time step *k_i_* and a total number of parcels *p_i_*. In order to conserve the wintering habitat, we define *B(t)*, the number of parcels to reserve in each wintering region at time *t* as a control variable. We represent this sequential conservation decision by a vector *B(t)=(b_1_(t), …,b_i_(t), …,b_n_(t))*. In other words *b_i_(t)* represents the number of parcels to reserve at time *t* in winter region *w_i_*. The current number of parcels already reserved for region *w_i_* at any time *t* is defined by $r_{i}(t)=\sum b_{i}(k)$ with *r_i_*(*t*) *≤ p_i_*. At each time-step, for each winter region *w_i_*, the sum of the number of parcels reserved *r_i_(t)*, number of parcels lost *l_i_(t)* and number of parcels still available *a_i_(t)* is equal to *p_i_* or more formally, $pi=ri(t)+li(t)+ai(t)$ for all *t* and all *i*.

The finite time-horizon *T,* is less than or equal to the terminal time. The terminal time is reached when no more parcels are available to reserve or be lost. Once parcels are reserved they are assumed to be protected and cannot be lost. Here we present the solution using a five year time-step with a finite time-horizon of 45 years, after which the terminal time is reached. Our first objective is to maximize the value *V(T,* ***N_w_****)* at time-horizon *T*, where *V(T,* ***N_w_****)* is defined as the number of redstarts protected in the winter regions *W*. Our second objective is to maximize the value *V(T,* ***N_w_*** *∧ x****N_s_****)* at time-horizon *T*, where *V(T,* ***N_w_*** *∧ x****N_s_****)* is the number of birds protected in winter region *W* and a proportion (e.g., *x=*0.3) of the total birds in each summer region *s_j_*, where the summer habitat is defined by a finite set of regions *S*={*s_1_, …, s_j_, …,s_m_*}.

With respect to migratory connectivity, the fraction of birds that travel from each winter region *w_i_* to each summer region *s_j_* is equal to *α_ij_*. For example, if 20% of the birds from winter region *w_i_* migrate to summer region *s_j_* then *α_ij_* = 0.2. The total number of birds *n_j_* in each summer region *s_j_* is the sum of the number of birds *n_i_* in each winter region *w_i_* multiplied by the fraction of birds *α_ij_* (Table S1) that migrate from each winter region *w_i_* to each summer region *s_j_* such that *n_j_*=.

Incorporating stochasticity makes the model computationally intractable; hence we keep the model deterministic. Using an optimal search algorithm, the optimal strategy for each objective is achieved by maximizing the scheduling strategy of *B(t)* in Equation 1 and 2 at time-horizon *T*:

*V*(*T,* ***N_w_****)* = (1)

*V*(*T,* ***N_w_*** *∧ x****N_s_****)* =, (2)

where *NBP_w_* and *NBP_s_* are vectors defining the number of birds per parcel for winter and summer respectively.

We consider ***N_w_*** (objective 1) and ***N_w_*** *∧* *x****N_s_*** (objective 2) as any possible goal states. The dynamic of the system can be expressed as follows, ***N_w_****’* and ***N_w_*** *∧**x****N_s_****’* are states with *b_i_(t)* reserves acquired and *d_i_*(*a_i_*(*t*) – *b_i_*(*t*)) parcels lost from region *w_i_*, with *d_i_*(*a_i_*(*t*) – *b_i_*(*t*)) equal to 0 if *a_i_(t)* equals 0, and 1 otherwise. We maximize these values while being subject to the constraint that the total cost of parcels acquired each year is less than or equal to the annual budget. We set the annual budget equal to the cost of the most expensive winter region *w_i_*:

(3)

where *c_i_* is the cost of acquiring a parcel from region *i* and *c_i_b_i_(t)* is the cost of acquiring *b_i_(t)* parcels.

**ii. Model parameters**

*Regions*

Because of the need to minimize the solution search space of this problem, we divided the winter sampling locations into 5 regions based on geographic proximity (Figure 1). Furthermore, hydrogen isotope analysis revealed that populations sampled within each of these regions showed similar patterns of connectivity to the breeding grounds [1]. Cuba was excluded from the analysis because we were unable to find reliable economic indicators necessary to estimate land cost [2]. For the breeding grounds, we used the 5 regions defined by Norris et al. (2006).

*Habitat loss*

Although American redstarts use a variety of non-breeding habitats, we chose to focus on coastal mangroves for two reasons. First, mangroves are one of the most threatened habitats in the tropics [3] and several species of migratory birds use this habitat during the non-breeding season. Second, estimates from other habitats were not available or reliable for all countries. While this decision limits our results, it should not influence our goal of testing if migratory connectivity can influence optimal conservation decisions. We used data from the Food and Agriculture Organization [4] to estimate the annual rate of loss of mangroves in each region and multiplied these rates by the total area of mangrove habitat in each region to estimate the amount lost annually.

*Habitat Parcel size and time-step*

To calculate the amount of mangrove habitat that is lost and that can be purchased in each time-step, we divided each region into parcels. To do this, we varied the parcel size per region by setting it equal to the rate of habitat loss in each region at each time-step. Larger parcels are, therefore, protected in regions that experience a higher rate of loss.

*Land Cost Function*

We derived a land cost estimate from the cost management function presented in Moore et al. [5], where log (Annual Cost, US$km^-2^ year^-1^) = 1.765 - 0.299log(Area, km^2^) + 1.014log(PPP) + 0.53log(GNI US$ km^-2^) - 0.771 * log(Area, km^2^) * log(PPP). This function calculates the ongoing management cost of a reserve and can be related to the purchase cost through the use of a factor by which the equation is multiplied [6]. By calculating the difference between the cost management function and the 2006 land cost from a sample of 105 real estate estimates, a multiplication factor for each region was developed (Western Greater Antilles, 101.7; Mexico, 99.5; Central America, 23.4; Eastern Greater Antilles, 159.7; Lesser Antilles/South America, 68.7). Gross economic indicators of Purchasing Power Parity (PPP) and Gross Net Income (GNI) were obtained from 2005 World Bank estimates [7]. PPP is a conversion factor which shows how much of a country’s currency is needed to buy a good worth $1 in the United States. GNI is the total value of goods and services (Gross Domestic Product) in addition to all interest and dividend payments received from other countries minus all interest and dividend payments to other countries.

*Budget*

At each five year time-step a fixed budget for habitat protection equal to the cost of a single parcel of land in the most expensive region was estimated at US$1.05 billion. This budget was equivalent to 1 parcel in Mexico, 8 parcels in Western Greater Antilles, 21 parcels in Central America, 1 parcel in Eastern Greater Antilles and 13 parcels in Lesser Antilles/South America.

*Bird density*

American redstart density was estimated through either line transects or point counts (Jamaica[8], Mexico P. Marra unpub. data; Bahamas, Puerto Rico, Panama, Florida, Trinidad/Tobago R. Norris unpub. data). Males do not sing on the non-breeding grounds but both sexes frequently produce distinctive ‘chip’ vocalizations throughout the non-breeding season. Density was estimated in all but three sites (Dominican Republic, Haiti and Belize). For Dominican Republic and Haiti, we used estimates from Puerto Rico, for Belize we used estimates from Panama. Density estimates for each of the five regions were averaged over sampling sites.

**iii. Solution search space**

Computing the number of possible solutions is equivalent to calculating the number of branches on a tree developed from the starting node (t=0) to the terminal nodes (t=T). Each node can potentially generate |A| new branches, or in our case decisions, therefore the number of solutions is equal to |A|^T^. The number of decisions |A| is a function of (i) the number of winter regions (ii) the number of parcels *p_i_* within each winter region *i* that are reserved *r_i_(t)* (iii) the number of parcels available for purchase *a_i_(t)*, and (iv) the amount of habitat that can be lost *l_i_(t)* within each region *i* and at each time-step. For our problem there are 49 possible decisions. Hence, for a 45-year time horizon and |A|=49, the size of the solution search space is 1.6e+15.

**iv. Model performance**

The difference in the estimated number of birds protected between the Dijkstra and ‘myopic’ algorithms was positively related to the specified time horizon (Figure S1). For the first 25 years, both algorithms performed equally, after which Dijkstra performed better. Myopic and Dijkstra’s algorithm converged to optimums at 35 and 55 years respectively.

**List of Supplementary Figures**

**Figure S1. Contrasting the performance of two algorithms** ‘myopic’ and Dijkstra over different time-horizons (5 to 60 years) showing the total number of birds saved when the objective function is to maximize the number of birds in the winter population.

**Literature cited**
