# Supplementary material for: Optimal Conservation of Migratory Species
Source: PLoS One. 2007 Aug 15;2(8):e751. doi: 10.1371/journal.pone.0000751 (PMC1937026; doi:10.1371/journal.pone.0000751)
Supplement: Table S1 — American redstart migratory connectivity. The proportion of birds that migrate from a winter region to each breeding region based on stable-hydrogen isotopes in feathers (Figure 1, see ref [1]). NW = Northwest; MW = Midwest; NE = Northeast; CE = Central-east; SE = Southeast. (0.04 MB DOC) [file pone.0000751.s002.doc]

**Table S1. American redstart migratory connectivity.** The proportion of birds that migrate from a winter region to each breeding region based on stable-hydrogen isotopes in feathers (Figure 1, see ref [1]). NW = Northwest; MW = Midwest; NE = Northeast; CE = Central-east; SE = Southeast.
