# Supplementary figures and images for: Optimal Conservation of Migratory Species
Source: PLoS One. 2007 Aug 15;2(8):e751. doi: 10.1371/journal.pone.0000751 (PMC1937026; doi:10.1371/journal.pone.0000751)

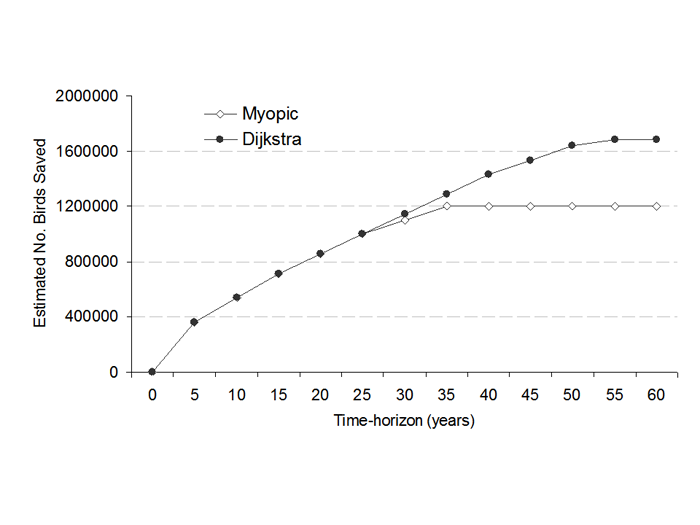

Supplement: Figure S1 — Contrasting the performance of two algorithms ‘myopic’ and Dijkstra over different time-horizons (5 to 60 years) showing the total number of birds saved when the objective function is to maximize the number of birds in the winter population. (0.08 MB TIF) [file pone.0000751.s003.tif]
